# Prediction paradigm involving time series applied to total blood issues data from England

**DOI:** 10.1111/trf.15705

**Published:** 2020-02-17

**Authors:** Anita K. Nandi, David J. Roberts, Asoke K. Nandi

**Affiliations:** ^1^ Big Data Institute, University of Oxford Oxford UK; ^2^ Radcliffe Department of Medicine, John Radcliffe Hospital National Health Service Blood and Transplant, Oxford Centre and BRC Haematology Theme Oxford UK; ^3^ Electronic and Computer Engineering Brunel University London Uxbridge UK

## Abstract

**BACKGROUND:**

Blood products are essential for modern medicine, but managing their collection and supply in the face of fluctuating demands represents a major challenge. As deterministic models based on predicted changes in population have been problematic, there remains a need for more precise and reliable prediction of demands. Here, we propose a paradigm incorporating four different time‐series methods to predict red blood cell (RBC) issues 4 to 24 weeks ahead.

**STUDY DESIGN AND METHODS:**

We used daily aggregates of RBC units issued from 2005 to 2011 from the National Health Service Blood and Transplant. We generated a new set of nonoverlapping weekly data by summing the daily data over 7 days and derived the average blood issues per week over 4‐week periods. We used four methods for linear prediction of blood demand by computing the coefficients with the minimum mean squared error and weighted least squares error algorithms.

**RESULTS:**

We optimized the time‐window size, order of the prediction, and order of the polynomial fit for our data set. The four time‐series methods, essentially using different weightings to data points, gave very similar results and predicted mean RBC issues with a standard deviation of the percentage error of 3.0% for 4 weeks ahead and 4.0% for 24 weeks ahead.

**CONCLUSION:**

This paradigm allows prediction of demand for RBCs and could be developed to provide reliable and precise prediction up to 24 weeks ahead to improve the efficiency of blood services and sufficiency of blood supply with reduced costs.

**ABBREVIATIONS**
MMSE: minimum mean squared error
NHSBT: National Health Service Department of Blood and Transplant
WLSE: weighted least squares error

Blood products are essential for modern medicine. Red blood cells (RBCs) are used widely in elective and emergency surgery, major trauma, hemorrhage, cancer care, and to support patients with congenital or acquired anemia.^1^ The call‐up of donors, scheduling of donor sessions, and manufacturing and supply of RBCs to hospitals must be coordinated to match demand. Managing the collection and supply of RBCs in the face of fluctuating demand on a daily, weekly, seasonal, and annual basis represents a major challenge for blood services. However, few planning tools and prediction models are available to allow precise and reliable prediction of demand. Any improvement of prediction tools would allow greater efficiency in the use of resources as well as a more resilient and secure blood supply chain.

Weekly demand for RBCs can change by 30% from week to week in our data set, and annual demand can change by 3% to 7% from year to year.^2^, ^3^ Predicting demand for RBCs in a simple deterministic model with the age structure of the population, the age‐specific incidence of disease, and the requirement of blood by indication and procedure for each disease has been attempted.^4^, ^5^, ^6^, ^7^ However, such models have considerable shortcomings and have proved to be unreliable, as they have underestimated the changes in medical and transfusion practice.^8^, ^9^, ^10^ Several predictions made with use of projected population growth and number and type of transfusion episodes overestimated demands.^5^ Major errors are introduced over short periods as the procedures used in each disease change, such as the introduction of less invasive surgery or the replacement of surgery by medical treatment for a specific disease. Furthermore, the indications and hemoglobin thresholds for transfusion have changed as evidence‐led patient blood management has improved patient care. These measures have reduced RBC transfusion per admission by improving patients' preoperative hemoglobin, lowered the hemoglobin threshold acting as a trigger for transfusion, and to some degree improved hemostasis and blood salvage. Such a wide variety of changes in medical and surgical management has perhaps made deterministic modeling highly prone to substantial errors.

An alternative strategy for prediction is to use time‐series methods where sequential elements of the series are hypothesized to be related by linear mathematical relations that can be estimated by some analysis of previous elements in the time series. Afterwards, the estimated respective coefficients can be applied to extend the series into the future. The use of time‐series methods for prediction have a long history.^11^, ^12^, ^13^, ^14^, ^15^ These methods are widely used in statistics, engineering, and the physical sciences. A variety of time‐series methods can be used.^16^ These approaches have proved to be successful in statistics,^17^ communications,^18^ signal processing,^19^ adaptive noise cancellation,^20^ earthquake prediction,^21^ mathematical finance,^22^ brain studies,^23^, ^24^ speech communication,^25^ weather forecasting^26^ and econometrics,^27^ to name a few examples. Although such methods can provide predictions successfully in several areas, they have not been applied systematically to the prediction of blood demands.

RBC usage or issue is readily available by day of issue. Usage of cells varies through the week and is less at weekends when elective surgery and transfusion of chronic anemia is reduced considerably. It is therefore more appropriate to use aggregated weekly data as the primary measure of RBCs use. In practice, the window for useful predictions of future demand are for 1 to 6 months to allow for matching of donor appointments and planning of donor sessions to predicted demand. Predictions at longer intervals, such as a year ahead, may be useful to match the overall collection capacity to predicted demand, particularly as demand falls. However, the accuracy of predictions over 6 months may limit their utility.

Here, we use four different time‐series methods to predict RBC usage 4 weeks to 24 weeks ahead and demonstrate that the mean RBC issues can be predicted with a standard deviation of the percentage error of 3.0% for 4 weeks ahead and 4.0% for 24 weeks ahead. The proposed paradigm may form the basis for reliable prediction of not only RBCs but also other components and even therapeutic procedures by blood services.

## MATERIALS AND METHODS

The focus of this paper lies in predicting the RBC usage from 4 to 24 weeks ahead with a novel paradigm for prediction incorporating different time‐series methods. Time‐series methods are a general set of techniques for predicting future values of a series of data that have some relationship to each other, specifically, where we can assume that the data values in the near future are related to the value of time points in the recent past. If this supposition did not have some truth, then variation from data point to data point would be random. If we have a long time series of data, we can reduce noise by smoothing the data with monthly demand instead of daily or weekly demand. This makes sense, as we need to adjust blood collection month by month as blood collection sessions are planned several months in advance.

The proposed paradigm is composed of three stages. The first stage is smoothing the weekly figures for issues. The second stage is detrending or applying a polynomial curve to the data. The third stage is time‐series modeling.

### Smoothing—data preparation

**Figure 1:**
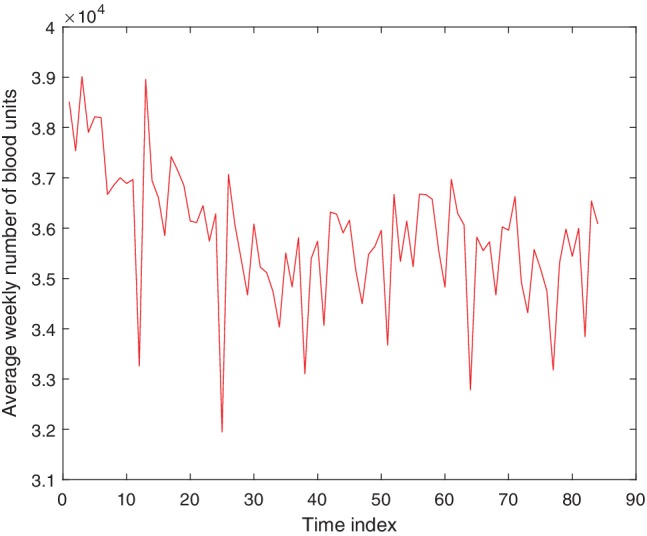
Average weekly blood usage for each non‐overlapping 4‐week period from February 2005 to July 2011. This data set contains 84 data points and is used for all prediction methods. The time index corresponds to the index of the 4‐week period. [Color figure can be viewed at http://wileyonlinelibrary.com]

Daily aggregates of RBC units used cover a period of 6.5 years from February 1, 2005, to July 31, 2011, and were obtained from the National Health Service Department of Blood and Transplant. On careful inspection of the real blood demand data it becomes clear that there are fluctuations both from day to day and from weekday to weekend. Therefore, in this stage, instead of daily data, we use aggregated data from 7 consecutive days or integer multiples of 7 consecutive days. This avoids effects of both daily variability and variability between weekdays and weekends. A new set of nonoverlapping weekly data was generated by summing the daily data over 7 days; that is, the first data point corresponds to the sum of Days 1 to 7, the second corresponds to Days 8 to 14, and so on. This new data set of weekly blood usage contains 338 data points. All the time‐series methods used nonoverlapping 4‐week data, as shown in Fig. 1. This was generated by summing the weekly data over 4 weeks and dividing by four, to give an average blood usage per week over that 4‐week period. In other words, the first data point is a weekly average blood usage over Weeks 1 to 4, the second is a weekly average over Weeks 5 to 8, and so on; this nonoverlapping 4‐week data set contains 84 data points.

### Detrending

**Figure 2:**
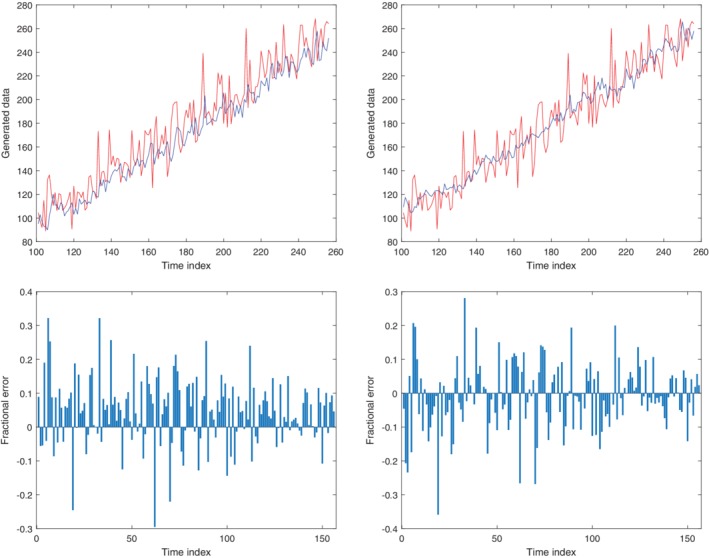
Simulated data of a straight line with noise (red) and predictions of the data with use of the MMSE method (blue). Results are shown with both the prediction method directly applied to the data (top left) and with the underlying trend removed prior to prediction (top right). Fractional error in the predictions with the prediction method directly applied to the data (bottom left), and the trend removed before prediction (bottom right) are shown. The time index corresponds to index of the simulated data. [Color figure can be viewed at http://wileyonlinelibrary.com]

Time‐series prediction methods are usually applied generically to a given data set. However, often time‐series data may contain an underlying trend, for example, a linear increase, in addition to other patterns and fluctuation due to noise. Preliminary experiments were performed with some manufactured data to investigate whether it would improve the prediction by removing the underlying trend before applying a time‐series prediction technique to the data. Data were generated according to a linear model, with unit gradient and vertical intercept of zero, with 10 dB of Gaussian noise added. Minimum mean squared error (MMSE) was used as the time‐series prediction technique in this preliminary study. Two predictions were performed, one in which no modification was made to the data before performing the prediction, and a second in which a second‐order polynomial was fitted to, and then subtracted from, the data to remove the trend before applying the prediction method. The results of both methods are shown in Fig. 2. It has been shown that removing the trend resulted in a significant improvement in the accuracy of the prediction.

**Figure 3:**
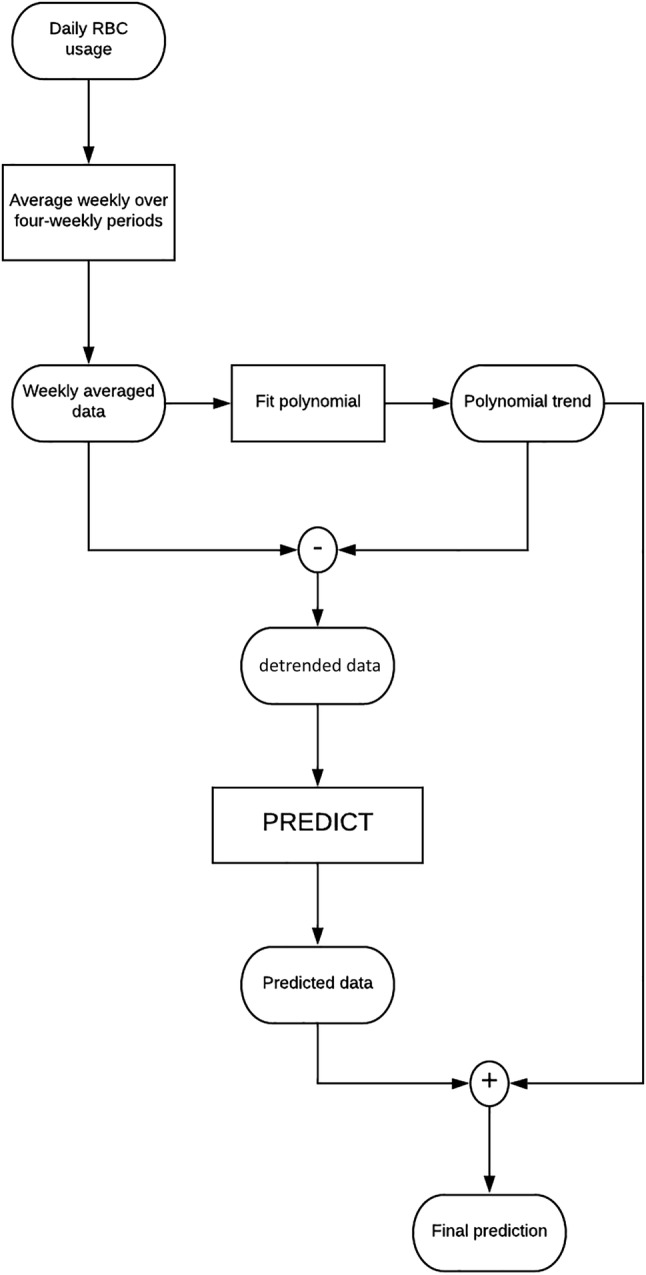
Schematic diagram of the processing steps involved in predicting future blood usage. Rounded boxes represent data, while rectangles represent a processing stage.

To apply the time‐series methods more effectively, the trend is first removed and the data adjusted to have a mean of zero. After the prediction has been performed, it is necessary to add the trend and mean back onto the result to calculate the predicted value. The trend is determined from a polynomial fit to the most recent *w* data points, where *w* is the time‐window size. Figure 3 shows a schematic of the steps taken to predict future blood usage.

### Time‐series methods

In this paper, four methods for predicting RBC usage are explored that focus around MMSE and weighted least squares error (WLSE), discussed in the following.

Time‐series prediction methods use a set of previous data points in the time series to predict future values. In general, it is assumed that the predicted value, $\hat{x}$, is some function of the past m values, as shown by

(1) $$\hat{x}\left(n+\alpha \;|\;n-1,n-2,\ldots ,n-m\right)=f\left(x\left(n-1\right),x\left(n-2\right),\ldots ,x\left(n-m\right)\right)$$

where *n* is the next time step in the series, *α* is the number of time steps ahead being predicted, and *x* is the data points in the time series. This defines *m* as the order of the prediction. In general, the function *f* is a nonlinear function of the variables, but in this paper, we restrict the function *f* to be a linear function of the variables; this is known as linear prediction, which is illustrated by

(2) x^n+α=∑i=1maixn−i

where *a*_*i*_ are a set of coefficients to be estimated. The error in this linear prediction, *e*(*n* + *α*), is defined to be

(3) en+α=xn+α−x^n+α

The linear time‐series prediction problem lies in investigating methods for determining the *a*_*i*_ coefficients. There are several algorithms for linear prediction techniques, that is, methods for computing the coefficients *a*_*i*_, that are well developed, for example, MMSE and WLSE.^12^, ^16^ However, there are circumstances when nonlinear data analysis methods are required. Machine learning algorithms can be used to develop nonlinear models for forecasting time‐series data.^28^, ^29^, ^30^, ^31^ Examples of these algorithms include kernel‐based machine learning, genetic programming, and artificial neural networks. Nonlinear prediction methods are equally valid for the time‐series data; however, they will not be considered in this paper.

First, MMSE provides an algorithm for determining the coefficients of the linear prediction based on minimizing the mean squared error, which is referred to as Method 1 here. Alternative methods based on the observation that the 4‐week data contain some large dips and peaks, aim to improve the prediction by mitigating the effect of these outliers. This can be achieved by creating weightings, *w*_*i*_, that favor those points closer to the polynomial fit. This way the large dips and peaks in the data will not have as much influence over the prediction. A polynomial *p*(*t*) is fitted to the data, *x*(*t*), so the chosen weightings will be some function of the difference between these values, *d*(*t*) = *x*(*t*) − *p*(*t*), where *t* represents time. The weightings are then normalized such that they sum to unity. Equation 4 is then used, after having found the weightings *w*_*i*_, to predict the next value:

(4) $$\begin{array}{c} \hat{x}\left(n\right)=\sum_{i=1}^{m}w_{i}x\left(n-i\right),\text{where}\;\sum_{i=1}^{m}w_{i}=1 \end{array}$$

where *t* = *n*T, with T being the sampling interval.

A control experiment needs to be provided to see if the chosen weightings improve the prediction. To provide this comparative measure, the coefficients were initially chosen to have equal weightings, that is, *w*_*i*_ = 1/*m*, which is referred to as Method 2. Later the weightings, *w*_*i*_, were chosen to be inversely proportional to ∣*d*(*n* − *i*)∣, which is referred to as Method 3. To see if the predictions can be further improved exaggerating these weightings, they were chosen to be inversely proportional to |*d*(*n* − *i*)|^2^, which is referred to as Method 4.

It is worth noting that time‐series modeling is a general method of predicting future values of a series of data that have some relationship to each other specifically, where we can assume that the data values in the near future are related to the value of time points in the recent past. In the formulations above, these relationships between future and past data are linear in all four time‐series methods. Thus, these are linear time‐series methods. Another alternative line of investigation can involve nonlinear time‐series methods, which is outside the scope of this paper.

### Figure of merit

Implementing each of the four time‐series methods described above gives a set of predictions, $\hat{x}\left(n\right)$, for each of their corresponding known true data values, *x*(*n*). The percentage error for each data point was calculated, $100\left(x\left(n\right)-\hat{x}\left(n\right)\right)/x\left(n\right)$. To assess quantitatively the accuracy of the prediction methods, the mean and the standard deviation of these percentage errors were calculated. Given that the mean percentage error is sufficiently small, it is more important that the standard deviation of the percentage errors is as small as possible; that is, the error in predictions does not vary by a large amount. Additionally, it is important to consider what proportion of the time the prediction is within a reasonable region around the true value. For the final results, we also quote the percentage of predictions that lie within the ±5% range of the true value.

## RESULTS

### Optimizing the parameters

The prediction paradigm, incorporating four time‐series methods, contains various parameters that can be altered, which would affect the accuracy of the prediction. These parameters include the time‐window size (*w*), the order of the prediction (*m*), and the order of the polynomial fit (*d*).

The time‐window size is the number of data points used to determine the value of the coefficients, *a*_*i*_, of the linear predictor. It is always the most recent *w* data points that are used, as they are going to give the best predictions for the near future. When deciding parameters such as the time‐window size, it is important to consider the nature of the time‐series data. In this case, the data are 4‐week data over 6.5 years, so we would expect some degree of periodicity over a year. This suggests that it would be beneficial to have a time‐window size that is a multiple of 13 (corresponds to a year in 4‐week data). To get an idea of the preferred time‐window size, the MMSE prediction was applied to the data using *w* = 13, *w* = 26, and *w* = 39. The results are shown in Table 1(a).

**Table 1:** (a) Varying time‐window size, w, fixing m = 5 and d = 2

*α*	Time‐window size, *w*		
	13	26	39
0	−0.02	0.28	0.03
	3.70	2.97	3.21
1	−0.05	0.08	−0.19
	4.13	3.00	3.20
2	−0.22	0.07	−0.20
	4.98	3.18	3.23
3	−0.19	0.06	−0.38
	5.54	3.21	3.13
4	−0.24	0.19	−0.56
	7.24	3.62	3.50
5	−0.07	0.19	−0.76
	8.99	4.02	3.69

(b) **Varying order, m, fixing w = 26 and d = 2**			
*α*	Order of the prediction, *m*		
	5	7	9
0	0.28	0.24	0.21
	2.97	2.85	2.94
1	0.08	0.07	0.11
	3.00	3.01	3.06
2	0.07	0.04	0.08
	3.18	3.19	3.28
3	0.06	0.08	0.07
	3.21	3.24	3.17
4	0.19	0.21	0.19
	3.62	3.65	3.43
5	0.20	0.22	0.24
	4.02	4.03	3.82

(c) **Varying order of polynomial fit, fixing w = 26 and m = 5**			
*α*	Order of the polynomial fit, *d*		
	1	2	3
0	0.48	0.28	0.28
	3.25	2.97	4.04
1	0.24	0.08	0.17
	3.13	3.00	4.17
2	0.24	0.07	0.16
	3.11	3.18	4.71
3	0.32	0.06	0.12
	3.16	3.21	5.12
4	0.36	0.19	0.23
	3.43	3.62	6.79
5	0.40	0.19	0.42
	3.65	4.02	8.30

Optimization of the prediction parameters: (a) time‐window size, w; (b) order of prediction, m; (c) order of polynomial fit, d. Results of MMSE prediction applied to blood usage data when predicting 4 weeks ahead (*α* = 0), 8 weeks ahead (*α* = 1), 12 weeks ahead (*α* = 2), 16 weeks ahead (*α* = 3), 20 weeks ahead (*α* = 4), and 24 weeks ahead (*α* = 5) for a range of parameter values are shown. In each box, corresponding to each experiment, the first number is the mean percentage error, and the second number is the standard deviation of the percentage errors.

Table 1(a) shows that *w* = 13 provides significantly worse predictions, so it can be ruled out. However, the difference between *w* = 26 and *w* = 39 is much less significant. As it is not clear that *w* = 39 provides a better prediction, a time‐window size of *w* = 26 was chosen to reduce computation.

The order of the predictor is the number of data points used in the linear prediction to explicitly calculate the next data point; that is, it is the number of terms in the sum given by Equation 2, which is equivalent to the number of coefficients, *a*_*i*_. To find the most suitable value of *m*, experiments were carried out using the different prediction methods. The prediction accuracy was investigated for *m* = 5, *m* = 7, and *m* = 9, and the results for MMSE prediction are shown in Table 1(b).

From Table 1(b) we can see that increasing the value of *m* makes very little difference to the quality of the prediction in any of the methods. Not only that, it is not clear from these results which value of m, if any, provides a better prediction. Therefore, we chose the smaller value of m to reduce the computational complexity. All the prediction methods were carried out using *m* = 5.

Before any of the prediction methods can be applied, the trend in the data must be removed, as shown above. This was done by fitting a polynomial and subtracting it from the data in each window. The aim of this process is to remove the general trend and leave the more frequent fluctuations. In one window there is not a large amount of variation in the gradient, that is, at most one turning point per window. This suggests that a large value for the order of the polynomial, *d*, would not be beneficial for the purpose, as it would start to attempt to fit the finer fluctuations in the data. To find the most effective value of *d*, some experiments were carried out using MMSE on 4‐week data after a polynomial fit was applied with values of *d* = 1, *d* = 2, and *d* = 3.

Here, we tried various orders of polynomials and decided what fits the data best by finding a balance between the orders of the polynomial and the errors from the fit. Table 1(c) shows that a polynomial fit of *d* = 2 provides the best predictions of the data. For all the prediction methods that use 4‐week data, a polynomial fit of order 2 was used to remove the trend.

### Comparison of the time‐series methods

**Figure 4:**
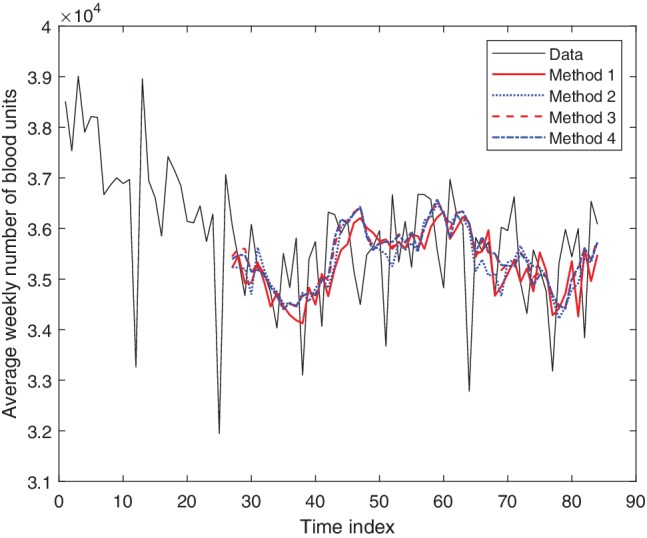
Results of the predictions using all four methods to predict the next 4‐week period. The data are shown in red. The time index corresponds to the index of the 4‐week period. [Color figure can be viewed at http://wileyonlinelibrary.com]

Each box in Table 2 shows the mean error, the standard deviation of the errors, as well as the percentage of predictions that lie within ±5% of the true value, for each of the four different prediction methods. For all quoted results, parameter values of *w* = 26, m = *5*, and *d* = 2 have been used. Predictions are made from one to six 4‐week periods ahead (i.e., 4‐week, 8‐week, 12‐week, 16‐week, 20‐week, and 24‐week). Plots of the predictions for 4 weeks ahead are shown in Fig. 4. The total blood usage data has been predicted for the next 4‐week period with a standard deviation in the error of 3.0%, with 95% of the predictions lying within 5%. The predictions for 24 weeks ahead achieve a standard deviation in the error of about 4.0%, with 85% of the predictions lying within 5% of the true value. The four different methods do not show much variation in performance.

**Table 2:** Results for each of the four prediction methods applied to blood usage data to predict 4 weeks ahead (*α* = 0), 8 weeks ahead (*α* = 1), 12 weeks ahead (*α* = 2), 16 weeks ahead (*α* = 3), 20 weeks ahead (*α* = 4), and 24 weeks ahead (*α* = 5)

*α*	Method			
	1	2	3	4
0	0.28	0.11	−0.16	−0.13
	2.97	2.96	2.92	2.90
	95	95	93	93
1	0.08	0.12	−0.18	−0.17
	3.00	2.99	2.97	2.99
	95	91	91	91
2	0.07	0.14	−0.18	−0.17
	3.18	3.19	3.11	3.13
	89	89	93	91
3	0.06	0.25	−0.10	−0.08
	3.21	3.28	3.11	3.11
	89	91	93	93
4	0.19	0.24	−0.15	−0.14
	3.62	3.79	3.61	3.65
	83	83	87	87
5	0.19	0.29	−0.19	−0.17
	4.02	4.39	4.12	4.12
	85	77	83	83

All these results use w = 26, m = 5, and d = 2. In each box, corresponding to each experiment, the first number is the mean percentage error, the second number is the standard deviation of the percentage errors, and the third number is the percentage of predictions that lie within ±5% of the true value.

As there are four different time‐series methods, for each data point there exist four different predictions. These can be combined by calculating the average of different prediction methods; this was found to show no significant improvement to the results.

## DISCUSSION

Here, we have evaluated our proposed prediction paradigm, incorporating four time‐series methods, to past RBC demand data to make predictions 4 weeks, 8 weeks, 12 weeks, 16 weeks, 20 weeks, and 24 weeks ahead. Each of the four used methods gave very similar results. This is the first published report of predictions of blood demand using time‐series data, and application of these methods may improve the effective planning of collection to the benefit of donors and blood services.

Method 1 provided predictions of aggregate demand for 4 weeks ahead with a standard deviation of 3.0%, with 95% of the predictions lying within 5% of the true value, and for 24 weeks ahead with a standard deviation in of 4.0% with 85% of the predictions lying within 5% of the true value. For predicting 4 weeks ahead, of the 5% of predictions that lie outside 5% of the true value, all predictions overestimate the blood unit demand. The maximum surplus for any individual prediction was 3331 blood units, while the maximum deficit was 1736 blood units. For predicting 24 weeks ahead, of the 15% of predictions that lie outside 5% of the true value, 4% overestimate demand (maximum surplus of 3964 units) and 11% underestimate demand (maximum deficit of 2809 units).

These margins of error would be operationally acceptable as the current average weekly issues of RBC units in England are approximately 27,000 units or 3800 units per day averaged over 9 months. The current stock levels of RBCs are currently maintained at between 8 and 10 days’ supply. Therefore, the blood supply chain could tolerate fluctuation in stock of 4000 units in any 1 week. In practice, adjustments to the supply could be made to cover such variation by minor changes to the collection schedule to maintain stable stock levels.

Previous attempts at predicting medium‐term demand for a group of patients or within a region or country have replied on simple linear extrapolation of year‐on‐year trend.^32^, ^33^ Generally, these methods have predicted a rising demand for blood based on demographics where the proportion of people over 75 years is rising, for example, in North America and Europe. In turn, these models generated concern about potential shortfall in the supply of blood from younger donors.^34^, ^35^ However, these attempts at medium‐term forecasting have been inaccurate and were unable to predict the trends in reduced blood demand due to changes in medical and surgical practice as well as patient blood management.^9^, ^36^ As a result, they have provided little firm evidence for planning, and short‐term planning has relied on time‐series methods from proprietary packages.

The improved prediction with reduced errors would allow greater efficiency in the call‐up of donors, scheduling of donor sessions, and manufacturing and supply of RBCs to match demand.

**Figure 5:**
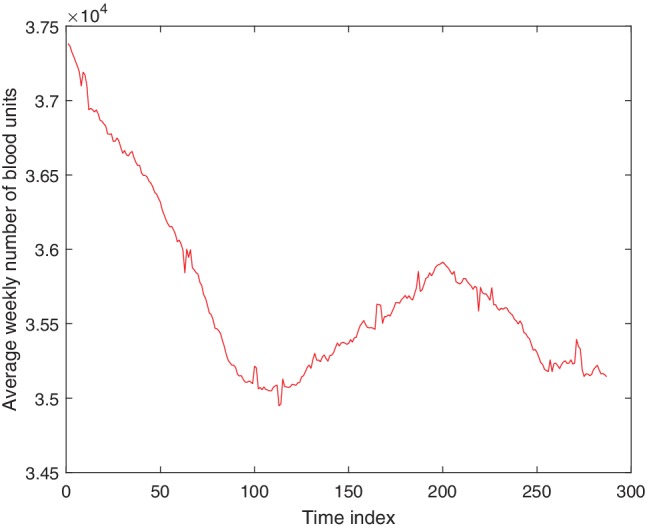
Average weekly blood usage for overlapping 52‐week periods, shifting by 1 week each time. This data set contains 237 data points. The time index corresponds to the index of the overlapping 52‐week period. [Color figure can be viewed at http://wileyonlinelibrary.com]

The time‐series demand forecasts described in this paper could be improved further. These methods have used 4‐week data. The series also have natural annual periodicity and smoothed‐overlapping data over a 52‐week period, shown in Fig. 5, may provide a better prediction by using the natural periodicity. All the methods in the paper have used the most recent data points to calculate the prediction. Another possible method would be to use the most recent data points considering the natural periodicities of the data. This way, the annual variations will be used rather than the local variation. Yet another avenue that could be investigated is nonlinear prediction.

Further improvements could be made if other information was available, for example, the changes in surgical procedure or practices in transfusion medicine and how they are being implemented in the different regions. There may indeed be other local or regional trends, although the usefulness of examining regional or subregional data is offset by the inherent increase in random errors as the numbers of units of blood decrease. Moreover, these prediction methods could be applied to each blood group (or category of blood groups) to provide group‐specific prediction of RBC use for more targeted call‐up of donors.

These findings of improved predictions using several time‐series methods that are tailored to the specific data sets potentially represent a significant advance in the techniques available to predict demand. If application of these methods and more reliable forecasts allow better matching of the resources needed to collect blood, there could be savings in marginal costs. Indeed, the improved predictions with reduced errors would allow greater efficiency in the call‐up of donors, scheduling of donor sessions, and manufacturing and supply of RBCs to match demand.

In conclusion, it is important to appreciate that a straightforward use of time‐series methods would not have produced results as good as those presented in this paper. The first two stages of smoothing and detrending make essential contributions to the success of the proposed paradigm. The proposed prediction paradigm, incorporating time‐series methods, appears to yield significant improvement in the accuracy of the prediction of blood demand with anticipated commensurate improvement in the effectiveness and efficiency of collection. These methods may be capable of significant improvements with more granular local data and by more precise alignment of the methods with the data.

## CONFLICT OF INTEREST

The authors have disclosed no conflicts of interest.
